# Ischemic Duodenal Ulcers Secondary to Superior Mesenteric Artery Stenosis in a Patient With an Aberrant Common Hepatic Artery: A Case Report and Review of the Literature

**DOI:** 10.7759/cureus.106781

**Published:** 2026-04-10

**Authors:** Imran Khokhar, Khaled Elsokary, Muhammad Haris Latif, Navanita Biswas, Ayesha Kang

**Affiliations:** 1 Internal Medicine, Reading Hospital, West Reading, USA; 2 Gastroenterology and Hepatology, Reading Hospital, West Reading, USA; 3 Internal Medicine, SSM Health St. Mary's Hospital, St. Louis, USA

**Keywords:** duodenal ischemia, hepatic arterial variant, ischemic duodenal ulcer, mesenteric ischemia, michel's type ix, replaced common hepatic artery, superior mesenteric artery stenosis

## Abstract

A 77-year-old male patient presented with persistent abdominal pain, nausea, vomiting, and diarrhea. Esophagogastroduodenoscopy (EGD) revealed duodenal ulcers suggestive of ischemia. Vascular imaging demonstrated a replaced common hepatic artery arising from the superior mesenteric artery (SMA), with the gastroduodenal artery originating from this SMA-dependent circulation, consistent with Michels' type IX anatomy. Significant stenosis at the SMA origin was identified. Endovascular stenting of the SMA resulted in symptom resolution and complete endoscopic healing of the ulcers. This case highlights the clinical importance of hepatic arterial variants and demonstrates how single-vessel disease can result in multi-territory ischemia when vascular anatomy is altered.

## Introduction

The gastrointestinal tract receives arterial supply from three major vessels: the celiac trunk, superior mesenteric artery (SMA), and inferior mesenteric artery [1]. The common hepatic artery typically arises from the celiac trunk and divides into the gastroduodenal and proper hepatic arteries [1].

Hepatic arterial variations are common, occurring in approximately 24-45% of individuals [2-4]. Michels' classification system describes 10 types of hepatic arterial anatomy [5]. In Michels' type IX, the entire common hepatic artery arises from the SMA, with a reported prevalence of approximately 2.8-4.5% [5,6].

Embryologically, these vessels develop from paired ventral segmental arteries connected by longitudinal anastomotic channels, and variations arise due to persistence or regression of these embryologic vessels [7,8].

The gastroduodenal artery (GDA) typically arises from the common hepatic artery and contributes to the pancreaticoduodenal arcade, which provides dual blood supply to the duodenum through connections with the SMA branches [9,10]. When both the common hepatic artery and the GDA arise from the SMA, SMA stenosis can reduce blood flow to both the liver and the duodenum.

## Case presentation

A 77-year-old man presented with nausea, vomiting, diarrhea, abdominal pain, and generalized weakness. Computed tomography (CT) demonstrated calcification of the SMA, raising concern for mesenteric ischemia.

The general surgery team performed a diagnostic laparoscopy, which did not reveal bowel ischemia. However, the patient continued to experience postprandial abdominal pain, food aversion, and diarrhea.

**Figure 1:**
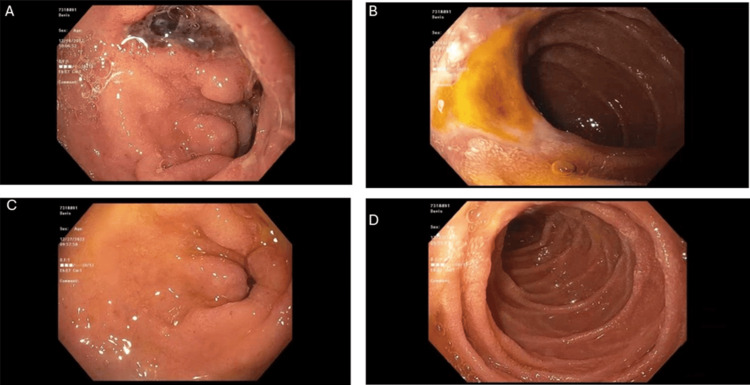
Endoscopic findings before and after superior mesenteric artery stenting (A, B) Initial esophagogastroduodenoscopy showed nonbleeding duodenal ulcers with a flat pigmented spot (Forrest class IIc), suspected to be ischemic in origin; (C, D) Follow-up esophagogastroduodenoscopy performed several weeks after stenting of the superior mesenteric artery demonstrated marked interval healing of the duodenal lesions.

Esophagogastroduodenoscopy (EGD) revealed Los Angeles grade D reflux esophagitis [11] without bleeding. The stomach appeared normal. Multiple duodenal ulcers with flat pigmented spots (Forrest class IIc [11]) were identified. Given the clinical context and imaging findings shown in Figure 1 below, these ulcers were suspected to be ischemic rather than peptic in origin.

Angiographic evaluation demonstrated a replaced common hepatic artery arising entirely from the SMA, with the gastroduodenal artery originating from this SMA-dependent circulation, consistent with Michels' type IX anatomy [5,6]. Severe stenosis was noted at the SMA origin.

Endovascular treatment was performed by placing a balloon-expandable stent at the SMA origin to restore blood flow [12,13]. Over the following weeks, the patient experienced complete symptom resolution. Repeat endoscopy confirmed healing of the duodenal ulcers.

## Discussion

Normal anatomy and embryological basis

The celiac trunk gives rise to the left gastric, splenic, and common hepatic arteries, forming the primary arterial supply to the foregut [1]. The duodenum receives a dual blood supply via the pancreaticoduodenal arcade, formed by branches of the GDA and SMA [9,10]. Embryologically, the celiac trunk and SMA arise from ventral segmental arteries, which are connected by longitudinal anastomoses, and variations occur due to the selective persistence of these channels [7,8,14].

Hepatic arterial variants

Hepatic arterial anatomy varies significantly, with only 55-76% of individuals demonstrating classical anatomy [2,15,16]. Michels' type IX represents a configuration in which the common hepatic artery arises entirely from the SMA [5,6], as shown in Table 1, based on a review of previous studies [3,5,6,16].

**Table 1:** Michels' classification of hepatic arterial variants CHA, common hepatic artery; RHA, right hepatic artery; LHA, left hepatic artery; LGA, left gastric artery; and SMA, superior mesenteric artery. In the present case, the patient had Michels' type IX anatomy, defined as the CHA arising from the SMA. The table was created by the authors using [3,5,6,16].

Type	Description	Percent
I	RHA and LHA from CHA	55%
II	Replaced LHA from LGA	10%
III	Replaced RHA from SMA	11%
IV	Replaced RHA and LHA	1%
V	Accessory LHA from LGA	8%
VI	Accessory RHA from SMA	7%
VII	Accessory RHA and LHA	1%
VIII	Accessory RHA and LHA and replaced LHA or RHA	4%
IX	CHA from SMA	4.5%
X	CHA from LGA	0.5%

Large angiographic and imaging studies have confirmed the spectrum and frequency of these variations [16,17].

Pathophysiology of ischemia

Chronic mesenteric ischemia typically requires stenosis or occlusion of multiple mesenteric vessels, given the extensive collateral circulation [18,19]. However, in this patient, both the hepatic artery and GDA originated from the SMA, meaning that a single proximal SMA stenosis compromised blood flow to multiple vascular territories.

Reduced perfusion through the GDA and pancreaticoduodenal arcade likely resulted in ischemic injury to the duodenal mucosa. Although uncommon, ischemic duodenal ulcers can present with abdominal pain and gastrointestinal bleeding [9].

Clinical implications

Hepatic arterial variants have important implications in hepatobiliary surgery, transplantation, and interventional radiology [2,15]. Injury to aberrant hepatic arteries can result in hepatic ischemia, necrosis, or abscess formation [8,15].

Preoperative identification using CT angiography (CTA) or magnetic resonance angiography (MRA) is critical for surgical planning and to avoid complications [3,16].

Management

Endovascular therapy is the preferred first-line treatment for chronic mesenteric ischemia due to lower morbidity and high clinical success rates compared to open surgery [18,19]. Balloon-expandable stents are particularly effective for ostial SMA lesions due to their high radial force and precise deployment [13].

In this case, SMA stenting restored perfusion to both hepatic and duodenal circulations. Resolution of symptoms and endoscopic healing confirmed vascular insufficiency as the underlying etiology.

## Conclusions

This case describes a rare presentation of ischemic duodenal ulcers caused by SMA stenosis in the setting of Michels' type IX hepatic arterial anatomy. In this configuration, a single-vessel lesion can compromise multiple vascular territories. Recognition of vascular variants and timely endovascular intervention are essential for appropriate management and prevention of complications. At the same time, there should be a detailed history, clinical examination, and investigations to rule out differential diagnoses of abdominal pain, while not forgetting to rule out uncommon diagnoses.
